# Responsive nanomaterials in biomedicine, patent path and prospect analysis

**DOI:** 10.3389/fbioe.2025.1539991

**Published:** 2025-02-04

**Authors:** Xinrui Liu, Hongmei Yuan

**Affiliations:** School of Business Administration, Shenyang Pharmaceutical University, Shenyang, China

**Keywords:** responsive nanomaterials, biomedicine, precision medicine, multimodal synergistic therapy, biomimetic design

## Abstract

In recent years, responsive nanomaterials have demonstrated tremendous potential in biomedical applications due to their unique advantages in precise drug delivery and controlled release. For complex diseases such as cancer, chronic inflammation, and genetic disorders, traditional treatment methods are often limited by insufficient targeting and significant side effects. Responsive nanotechnology, by sensing specific internal or external stimuli, has significantly enhanced the precision and efficiency of treatments. This study systematically summarizes the technological trajectory and emerging research directions of responsive nanomaterials through global patent and literature data, employing main path analysis, derivative path analysis, and keyword co-occurrence analysis. The results reveal the evolution of this field, from the optimization of early single-stimulus-responsive nano delivery systems to the rise of theranostics integration, followed by advancements in multi-stimuli-responsive synergistic therapies, and finally, the innovation in biomimetic material design. Each developmental phase has increasingly focused on adapting to complex biological environments, achieving superior targeting performance, and enhancing therapeutic efficacy. Keyword co-occurrence analysis highlights key research hotspots, including biomimetic design, multimodal synergistic therapies, and emerging response mechanisms. In the future, responsive nanomaterials are expected to play a pivotal role in personalized medicine, multifunctional carrier design, and complex disease management, providing novel insights and technological support for precision medicine.

## 1 Introduction

**FIGURE 1:**
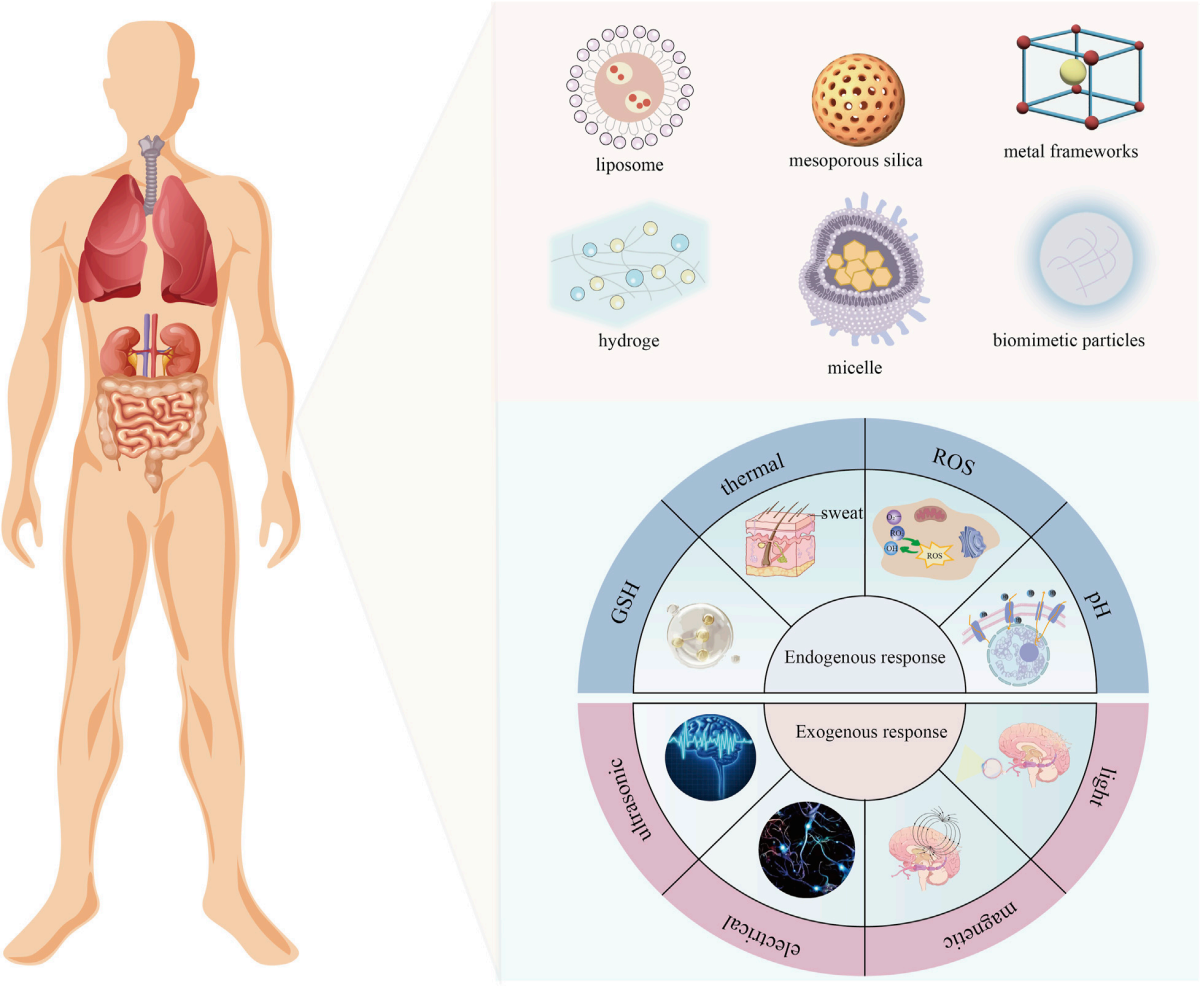
Responsive nanomaterials and stimulation means.

In recent years, the biomedical field has faced significant challenges in addressing complex diseases such as cancer (Fang et al., 2020), chronic inflammation (Ma et al., 2020), and genetic disorders. These diseases are often associated with complex pathologic environments and multifactorial-driven processes of lesion formation. Traditional therapeutic approaches, such as chemotherapy and targeted drugs, have achieved some success. However, due to the lack of selectivity to the lesion microenvironment, their therapeutic efficiency is often limited and accompanied by severe side effects and drug resistance problems (Wang et al., 2024c). Consequently, achieving precise drug delivery, controlled release, and targeted action at pathological sites has become a central challenge in modern biomedical research. Responsive nanomaterials (Figure 1), as a class of intelligent materials capable of sensing specific stimuli and undergoing functional transformations, offer highly attractive solutions to these issues (Wang et al., 2024c).

To address these complex therapeutic demands, current research on responsive nanomaterials primarily focuses on three key directions. The first is the design of specific responsive nanomaterials tailored to various stimulus signal systems. The second involves enhancing the overall performance of responsive nanomaterials by optimizing their structural properties or incorporating synergistic theranostic strategies. The third is applying the designed responsive nanomaterials to diverse biological scenarios. Centered on these three core research directions, responsive nanomaterials have demonstrated diverse functionalities and extensive application potential.

Taking the tumor microenvironment as an example, responsive nanomaterials are employed to develop pH-responsive nanomedicine delivery systems based on the pH differences between tumor and normal cells (the tumor microenvironment typically has a pH range of 6.4–6.8, whereas normal blood and tissue pH is approximately 7.4) (Guo et al., 2024). These systems enable precise drug release in tumor-specific pH environments while maintaining stability in normal tissue cells (Wike-Hooley et al., 1984; Koltai, 2020; Qin et al., 2023). Similarly, leveraging the elevated concentration of GSH in tumor cells (∼2–10 mM) compared to the extracellular environment (2–20 μM), reduction-responsive drug carriers have been developed to regulate drug release in the tumor microenvironment (Cui et al., 2023). Exploiting enzyme activity and concentration gradients at tumor sites further facilitates triggered drug release (Hu et al., 2012; de la Rica et al., 2012; Zeng et al., 2022a). Additionally, elevated levels of reactive oxygen species (ROS) provide precise triggering conditions for drug release in atherosclerotic lesions (Luo et al., 2023). External stimuli-responsive nanomaterials further facilitate non-invasive therapies (El-Sawy et al., 2018) such as photodynamic therapy (PDT). (Cardano et al., 2018), photothermal therapy (PTT) (Lee et al., 2015), and magnetically targeted therapy (Klostergaard and Seeney, 2012).

To further enhance the adaptability and efficacy of responsive nanomaterials in complex biological environments, researchers have conducted extensive studies on design and synthesis strategies. These efforts include the development of high-performance materials through various preparation methods and the integration of chemical modifications, self-assembly techniques, and intelligent design strategies to improve drug loading capacity, response sensitivity, and *in vivo* stability. Carrier materials such as liposomes (Xue et al., 2012), micelles (Xue et al., 2012), inorganic nanoparticles (NPs), metal and metal oxides (Yu et al., 2017), and metal-organic frameworks (MOFs) (Pei et al., 2023; Fatima et al., 2024) have attracted significant attention from the scientific and medical communities due to their potential for remote-controlled applications (Mu et al., 2019). These optimizations and advances in therapeutic strategies have enabled responsive nanomaterials to demonstrate significant efficacy in addressing tumors, inflammation, and neurodegenerative diseases. For instance, Yan proposed a GSH-responsive nanodrug delivery system for melanoma treatment. Using a carboxymethyl chitosan carrier combined with the chemotherapeutic drug camptothecin and the photosensitizer Rhodamine B, this system achieved synergistic effects of chemotherapy and PDT (Yan et al., 2024). Similarly, CN114983930A (Zhao et al., 2022) described a ROS-responsive brain-targeted nanogel dual-release system based on polymer hydrogels. The system integrated ROS-responsive nanoparticles with thermosensitive hydrogels, focusing on treating oxidative stress-related brain disorders such as depression.

Technology trajectory refers to the development patterns or pathways exhibited under a specific technological paradigm to address technical challenges (Wei et al., 2024). Analyzing technology trajectories enables researchers to uncover the underlying logic and key milestones of technological advancements, providing a scientific basis for achieving innovation (Han et al., 2024). In recent years, technology trajectory analysis has been widely applied in frontier fields such as biomedicine and new materials. For example, Kim conducted a main-path analysis of chromatography technology patents from 1970 to 2018, dividing the development of chromatography technology into four stages: the first stage focused on the development of sample injection and flow rate control systems; the second stage introduced computer technology to achieve system automation; the third stage aimed at improving column separation speed and efficiency; and the fourth stage, centered on HPLC and UPLC, enhanced system stability and separation performance (Kim et al., 2020). The study reveals the evolutionary path of chromatography technology, providing insights for technological innovation and industrial upgrading. Similarly, this analytical approach has been applied to the field of nano delivery technologies. Wei constructed a patent citation network for inorganic nanomaterials in cancer applications (Wei et al., 2024). The main path analysis revealed that key technologies for inorganic nanomaterials in cancer diagnosis and treatment include biological detection and imaging, drug delivery, phototherapy, magnetic hyperthermia, and radiotherapy, with a gradual shift toward theranostics integration. Therefore, by mapping technology trajectories, researchers can trace the origins, critical time periods, and breakthroughs in responsive nanomaterial research, providing scientific support for pharmaceutical enterprises or governments in developing strategic plans and making informed decisions.

In the past, most studies about responsive nanomaterials have focused on literature reviews, with relatively few in-depth analyses at the patent level. For example, Raza summarized the design and development of various redox-responsive drug delivery systems, including liposomes, micelles, nanoparticles, nanogels, and prodrug-based nanomedicines, with a focus on their applications in tumor-targeted drug delivery (Raza et al., 2018). Song systematically reviewed recent advances in endogenous and exogenous stimuli-responsive nanodrug delivery systems for atherosclerosis treatment and categorized these systems into organic, inorganic, and multifunctional composite nanodrug delivery systems (Song et al., 2022). While literature reviews comprehensively summarize existing research outcomes, they often emphasize theoretical summaries and fundamental research progress. In contrast, patent analysis offers stronger practical application value and technological foresight. The key information contained in patents can reveal the intrinsic logic of technological development, technical layout, and core technological nodes (Wang et al., 2024d). However, systematic studies leveraging patent information to analyze innovation and evolution in the responsive nanomaterials field from a technology trajectory perspective remain scarce, particularly those employing main path and derivative path methods. By analyzing the core and branching paths of patent technologies, researchers can further uncover overall trends in technological progress and deeply explore the interactions and evolutionary patterns among different technological entities. This provides valuable references for future development in the responsive nanomaterials field.

This paper aims to systematically review the technological evolution and application potential of responsive nanomaterials in the biomedical field, providing a comprehensive analysis of their current state and future trends. By integrating global patent data, the main and derivative paths were mapped to trace the evolution of core technologies and identify innovation directions for key technologies. At the same time, to ensure data diversity, the study also incorporates recent literature and employs keyword co-occurrence analysis to generate heatmaps, uncovering research hotspots and potential development directions. The organic integration of patent data and literature not only offers a multidimensional understanding of existing technological layouts but also provides robust support for academic research on responsive nanomaterials in the biomedical field, fostering their clinical applications.

## 2 Methods

### 2.1 Data collection process

The data for this study comprise two parts: patent data and literature data. The patent data was sourced from the Derwent Innovations Index (DII) database, where a comprehensive screening of keywords in patent titles and abstracts (TIAB) was conducted. We did not limit the start date of the patent data samples and used the date of the start of the study (2 September 2024) as the end date for sample retrieval. The keywords used were “nano” and “responsive,” combined with the International Patent Classification (IPC) to focus on the biomedical field, specifically IPC classification numbers A61P, A61K, C07K, C07H, C12N, C12Q, and G01N (e.g., A61K stands for specific preparations for medical, dental, or toiletry purposes). Literature data were retrieved from the Web of Science Core Collection, restricted to publications from 2023 to 2024, using the keywords “nano,” “drugs or genes,” “delivery” and “responsive.” After manual screening and data cleaning, a total of 4,112 patents and 963 literature records were retained.

### 2.2 Main and derivative path analysis

In 1989, Hummon and Doreian first proposed the main path analysis method, which uses citation information from academic papers or patents to trace the evolution of major ideas in a scientific field (Hummon and Dereian, 1989). The core concept of main path analysis is based on a citation network perspective, treating scientific publications or patents as nodes in a network and linking these nodes through citation relationships to map the paths of knowledge dissemination. Further, to address the limitations of traditional main path analysis, which may overlook some key information, subsequent studies improved the method. Kim and Shin proposed the Main Path and Derivative Path Analysis method, an extension that not only identifies primary technological trajectories but also captures additional branching paths, revealing a broader framework of technological development (Kim and Shin, 2018). Therefore, patent-based analysis of the main path and derivative path can provide a more comprehensive depiction of the responsive nanotechnology evolution’s multilayered structure.

### 2.3 Keyword co-occurrence heatmap analysis

In 1988, Law proposed the co-occurrence analysis method, which encompasses co-citation and co-word analyses (Law et al., 1988). The keyword co-occurrence analysis is employed in this study. Each published document may contain 5–6 author keywords relevant to the paper. By constructing a keyword co-occurrence matrix from the selected literature, a co-occurrence network can be established. Furthermore, the network can be visualized using a heatmap with the help of VOSviewer. In the heatmap, points represent keywords, and their positions and distributions are determined by the co-occurrence relationships among the keywords. The color and brightness of the points indicate the research intensity or concentration associated with a particular keyword. Therefore, the heatmap based on keyword co-occurrence analysis provides an intuitive representation of the high-frequency keywords’ distribution and interrelations in the responsive nanotechnology field.

## 3 Results and discussion

### 3.1 Main and derivative path analysis results

**FIGURE 2:**
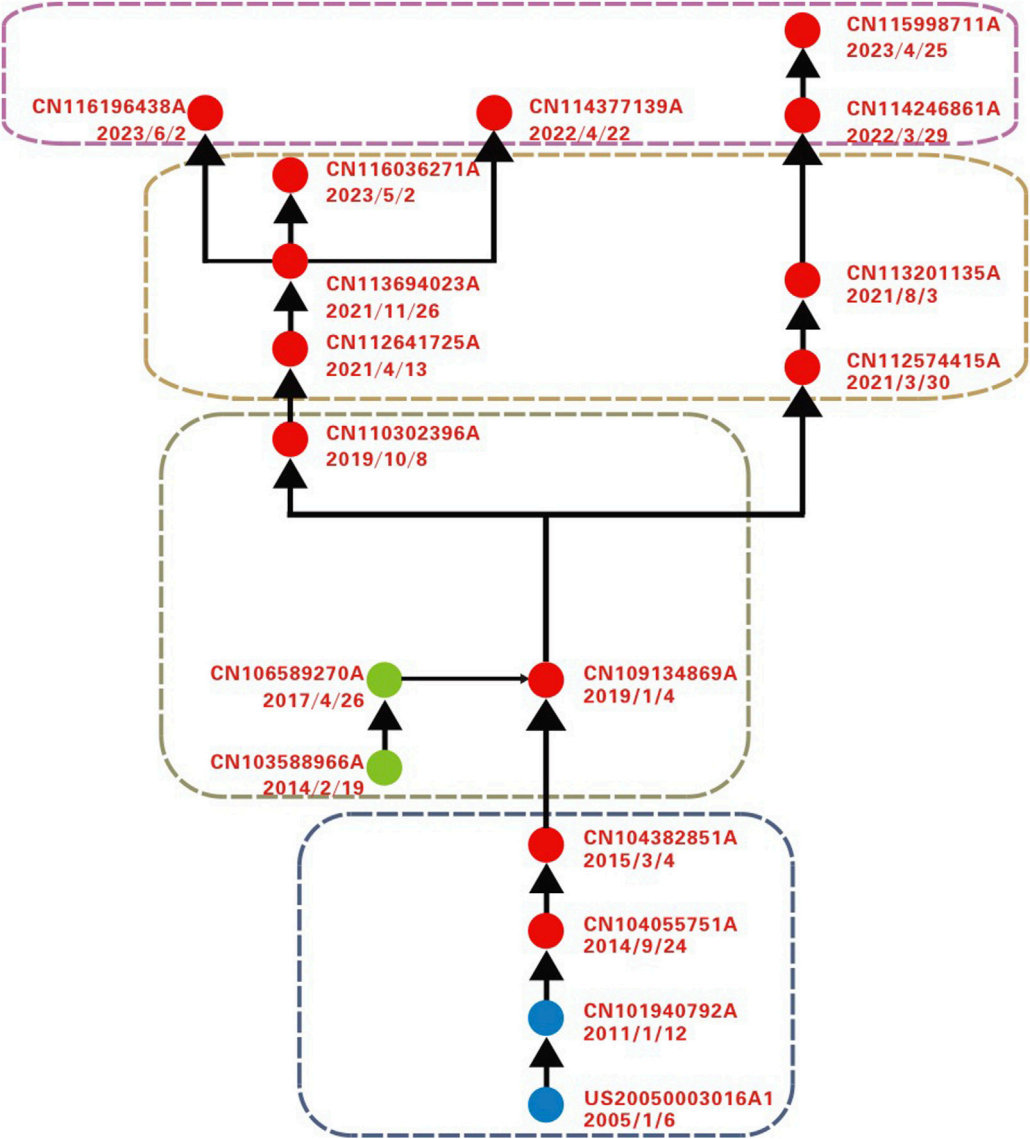
Technological trajectory of responsive nanomaterials on main and derivative path analysis.

By constructing a patent citation network and visualizing it using Pajek, the final main and derivative paths were identified (as shown in Figure 2). Red nodes represent the main path, while blue and green nodes indicate derivative paths. The patent information for each path node is detailed in Table 1. Analysis reveals that different types of responsive nanomaterials have been applied in fields such as tumors, atherosclerosis, and cardiovascular diseases, particularly in biological imaging and theranostics integration. The overall development trend transitions from theranostics integration toward combination therapy and biomimetic drug delivery systems.

**TABLE 1:** Main and derivative path node information.

Patent number	Title	Applicant	Publication date
US20050003016A1	Controlled release polymersomes	Discher Dennis E; Ahmed Fariyal	2005-1-6
CN101940792A	PCL-b-PEG-b-PCL carried hydrophobic medicine polymer vesica as well as preparation method and application thereof	Institute of Biomedical Engineering, Chinese Academy of Medical Sciences	2011-1-12
CN104055751A	Long-circulating and targeting synergistic multifunctional anti-tumor targeting nano-drug carrier	Nankai University	2014-9-24
CN104382851A	Method for preparing intelligent target medicine carrying composite micelles	Nankai University	2015-3-4
CN109134869A	Hydrogen peroxide responding type targeted fluorescent medicine-carrying nanomaterial and preparation method	Jilin University	2019-1-4
CN110302396A	Multifunctional liposome based on hydrogen peroxide response and preparation method and application thereof	Jilin University	2019-10-8
CN103588966A	Preparation method of amphiphilic copolymer for targeted photodynamic therapy	Jiangsu University	2014-2-19
CN106589270A	Preparation method of star polymer-based drug carrier material with fluorescence labeling and temperature responsiveness	Tongji University	2017-4-26
CN112574415A	Active oxygen responsive material as well as preparation method and application thereof	Jilin University	2021-3-30
CN113201135A	Preparation method and application of active-oxygen-responsive material PAM-SH	Jilin University	2021-8-3
CN112641725A	Targeted nano-micelle as well as preparation method and application thereof	Jiangsu University	2021-4-13
CN113694023A	Oxidation response type nano micelle and preparation method and application thereof	Wuhan University Zhongnan Hospital	2021-11-26
CN116036271A	A nanoprodrug for ultrasound-responsive release of nitric oxide gas and camptothecin thereof and preparation method and application thereof	Peking University Third Hospital (Peking University Third Clinical Medical College)	2023-5-2
CN114246861A	The invention relates to a preparation method of drug-loaded nanoparticles with shear stress response	Jilin University	2022-3-29
CN115998711A	A targeted nanodrug delivery system for reversing tumor drug resistance and its preparation method and application	China Pharmaceutical University	2023-4-25
CN114377139A	Carriers, drug delivery systems and uses thereof	Sichuan Academy of Medical Sciences Sichuan Provincial People’s Hospital	2022-4-22
CN116196438A	Preparation method of oxidation-responsive nanopreparations	Wuhan University of Science and Technology	2023-6-2

#### 3.1.1 Phase I: responsive nanodelivery systems based on traditional optimization

The design of responsive nanocarriers is an optimization and improvement based on traditional nanodelivery materials (Shi et al., 2017). For example, patent US20050003016A1 introduced a controlled-release system based on amphiphilic block copolymers (Discher and Ahmed, 2005). The core materials, including PEG-PLA (polyethylene glycol-polylactic acid) or PEG-PCL (polyethylene glycol-polycaprolactone), were designed with a hydrophilic group volume fraction of 20%–50%, resulting in the construction of stable vesicle membranes with self-healing properties. This system leverages the hydrolytic triggering properties of the materials to regulate drug release rates, serving as a classic example of early chemically responsive materials. In comparison, patent CN101940792A focused on improving the delivery efficiency of hydrophobic anticancer drugs. Using a thin-film hydration method, polymer vesicles were prepared by combining hydrophobic drugs with copolymers (e.g., PCL-b-PEG-b-PCL) (Zhang and Song, 2011). This design not only extended the *in vivo* circulation time of the nanocarriers but also utilized the enhanced permeability and retention (EPR) effect to achieve effective drug accumulation at tumor sites. US20050003016A1 and CN101940792A respectively provide unique approaches to responsive mechanisms and long-circulation structures. The former emphasizes controlled drug release via chemical degradation through hydrolytic responsiveness, while the latter combines long-circulation characteristics with the EPR effect to facilitate efficient drug accumulation at target sites. The integration of these technologies offers promising directions for the design of diverse responsive nanocarriers.

In further research, patent CN104055751A developed a nano-drug delivery system combining long circulation and targeted synergistic effects (Shi et al., 2014). This system forms a core-shell composite micelle through the self-assembly of PEG-b-PCL and PCL-b-PAE-c (RGDfK) block copolymers in the aqueous solution. By integrating the long-circulation properties of PEG surface modification with the functionality of targeted groups, the system successfully transitions from passive targeting based solely on the EPR effect (e.g., CN101940792A) to a combination of passive and active targeting. Patent CN104382851A further enhanced the carrier’s intelligence by introducing a dual temperature- and pH-responsive targeted drug delivery micelle (Yuan et al., 2015). This system utilizes the reversible phase transition of thermosensitive polymers to enable reversible shielding and deshielding of targeting ligands in blood circulation and tumor hyperthermia regions. In blood circulation (pH 7.4, 37°C), the targeting ligands are shielded to reduce mononuclear phagocyte system clearance. At tumor sites (slightly acidic, 40°C–44°C), the ligands are deshielded to restore activity. This reversible “shielding-deshielding” mechanism significantly improves the carrier’s adaptability to complex physiological environments.

In this stage of research, beyond the pH- and temperature-responsive nanomaterials highlighted in the path analysis, other stimuli-responsive nanomaterials, including light-responsive, enzyme-converting, and GSH-responsive systems, have also become research hotspots. For example, patent KR1020140103426A developed a light-responsive nanocomposite material based on polysaccharides and its preparation method. By grafting HMNB (4-(4-(1-hydroxyethyl)-2-methoxy-5-55 nitrophenoxy) butyric acid) onto polysaccharides, the system forms a nanocomposite material capable of structural changes under light exposure, enabling controlled drug release and targeted delivery, particularly for tumor chemotherapy (Lee and Park, 2014). The material, with a particle size of 50–200 nm, efficiently encapsulates drugs (e.g., paclitaxel and doxorubicin) and uses light to trigger drug release, thereby reducing side effects and improving therapeutic outcomes. Similarly, patent CN104152433A proposed a glucose-responsive microtubule-kinesin transport system (Li et al., 2014). This system assembles glucose oxidase microspheres or microcapsules with ATP synthase, using energy generated from glucose oxidation to control the movement of kinesin-driven microtubules without the need for external ATP and achieving ATP regeneration. This system exhibits glucose responsiveness, with microtubule motion activated in glucose-rich regions and stationary otherwise, allowing precise control over the transport process and enabling the delivery of various substances. Patent CN111714456A introduced a multifunctional nanovesicle drug delivery system with both targeting and fluorescence-tracing capabilities (Pei et al., 2020). Using mannose-functionalized pillar [5] arenes and disulfide-bridged dicyanomethylene-4H-pyrans, the vesicle achieves targeted transport, controlled release of anticancer drugs, and fluorescence tracking. In high-GSH environments, the disulfide bonds cleave, triggering drug release and activating fluorescence tracking. This system significantly enhances anticancer efficiency, reduces side effects, and offers a novel approach to anticancer drug delivery and monitoring.

#### 3.1.2 Phase II: from drug delivery to theranostics integration

In the next stage, patent CN109134869A developed an intelligent nanodrug delivery material with a hydrogen peroxide (H₂O₂)-responsive mechanism (Li et al., 2019a). By reacting statin drug precursors with oxalyl chloride, combining them with PHEMA (polyhydroxyethyl methacrylate), and coupling macrophage-targeting molecule ISO-1 and fluorescent polyethylene glycol via reagents such as dicyclohexylcarbodiimide, a multifunctional intelligent carrier was successfully constructed. This system integrates H₂O₂ responsiveness, fluorescent visualization, and macrophage-targeted therapy, offering a multifunctional theranostic integration solution for atherosclerosis. For a more precise response mechanism, patent CN110302396A developed a multifunctional liposome based on H₂O₂-responsive, along with its preparation method and applications (Li et al., 2019b). The liposome, composed of phosphatidylserine and the diblock copolymer PEG-PPS (polyethylene glycol-polypropylene sulfide), encapsulates dual-modality probes (e.g., FITC-SiO2@Fe3O4 and MRI) and combines H₂O₂-responsive properties to simultaneously achieve disease imaging and drug therapy. The integration of MRI and fluorescence imaging improves diagnostic accuracy and enhances imaging depth. MRI imaging is particularly suitable for accurately imaging deep lesions such as atherosclerotic plaques due to its superior penetration ability, which compensates for the limited penetration depth of fluorescence imaging.

During the same period, responsive nanomaterials for tumor theranostics integration also made significant progress. Patent CN103588966A developed an amphiphilic copolymer for targeted photodynamic therapy and its preparation method (Dai and Wang, 2014). This method utilized porphyrin as the core, combined with polylactic acid and lactose polymers, to produce hybrid biomaterials composed of star-shaped polylactic acid-lactose block copolymers via a mild RAFT (reversible addition-fragmentation chain transfer) polymerization. By precisely controlling the molar ratio of sugar monomers to the polylactic acid macroinitiator, the material’s performance was significantly enhanced. The sugar molecules specifically bind to receptor proteins on the cell surface, enabling precise drug release at tumor sites. Although this patent demonstrated excellent targeting and safety, its application is limited by the light penetration depth and specific light source requirements, making it more suitable for treating superficial cancers such as skin cancer. To overcome these limitations, the introduction of temperature-responsive mechanisms has provided new pathways for treating deep-seated tumors. Patent CN106589270A developed a temperature-responsive star-shaped polymer drug carrier (Ren et al., 2017). This carrier was created using ATRP (atom transfer radical polymerization) to introduce hydrophobic biodegradable polyester chains into the star-shaped polymer, followed by grafting with poly (2-hydroxyethyl methacrylate) and completing fluorescent labelling through the reaction of fluorescent small molecules with hydroxyl groups. This design not only achieves targeted delivery to deep-seated lesions but also integrates diagnostic and therapeutic functions, paving new directions for tumor theranostics integration.

#### 3.1.3 Phase III: evolution from single response, multiple response to synergistic treatment

As previously mentioned, patent CN109134869A marks a significant milestone in the design of responsive nanomaterials and theranostics integration (Li et al., 2019a). This patent introduces oxidative stress environments (H₂O₂) commonly found in specific pathological tissues (such as tumors, inflammation, or atherosclerotic plaques) as a trigger, offering a more intelligent and fluorescently traceable response mechanism compared to the widely used environmental factors like pH and temperature. Due to these characteristics, this key development has the potential to further expand its applications in the treatment of atherosclerosis, tumors, and theranostics integration.

Patent CN112574415A introduces the design, synthesis, and application of ROS-responsive materials (Ren et al., 2017). PGED (ethylenediamine open-loop poly glycidyl methacrylate) was synthesized via a copper chloride-catalyzed polymerization reaction, followed by a reaction with thioacetate to yield PGED-PPS (polyglycidyl methacrylate-polysulfide propylene), which formed nano micelles when combined with antithrombotic drugs (such as simvastatin). The nano micelles dissolve under ultrasonic treatment and are subsequently obtained through dialysis, exhibiting targeted drug-release properties. The PGED-PPS material responds to H₂O₂ and scavenges ROS, working synergistically with simvastatin to achieve a highly effective antithrombotic treatment. Subsequently, Patent CN113201135A further enhances the performance of ROS-responsive materials by introducing a preparation method for PAM-SH and its application (Li et al., 2021). Using PAMAM (polyamide-amine) dendrimers as the core, PAM-SH was synthesized by incorporating sulfhydryl (SH) groups and self-assembling with simvastatin acid (SA) to form nanoparticles. The nanoparticles can precisely release drugs in regions with elevated ROS concentrations, such as atherosclerotic plaques, while simultaneously providing dual therapeutic effects through ROS scavenging, including antioxidant and antithrombotic actions.

From Patent CN109134869A to CN112574415A, and then to CN113201135A, the gradual innovation and development of ROS-responsive nanomaterials in drug delivery systems can be observed. This technological approach has evolved from a single-response function to a design that integrates dual and multifunctional synergies, with each generation of patents introducing new functions or optimizing existing properties to achieve more precise and efficient therapeutic outcomes. This research trajectory has also been validated in cancer therapy.

In another approach, Patent CN112641725A expands the application of ROS-responsive nanomaterials in cancer therapy, integrating sonodynamic therapy (Shen et al., 2021). PEG serves as the hydrophilic shell, while PPS acts as the hydrophobic core. The resulting amphiphilic block copolymer, PEG-PPS, self-assembles into nanomicelles and encapsulates a sonosensitizer (such as hypocrellin). Under the dual action of ROS and ultrasound, the micelles can precisely release the drug, enabling effective cancer treatment. Patent CN113694023A provides an amphiphilic ROS-responsive nanodrug delivery system (Deng et al., 2021). The system connects the hydrophobic antitumor drug (such as doxorubicin) to the nanomicelles via a thioketal linkage with mPEG (polyethylene glycol monomethyl ether). Under sonodynamic therapy, the sonosensitizer generates a large amount of ROS, which stimulates the cleavage of the thiosulfonate linkage, thereby releasing doxorubicin. This mechanism significantly improves the drug enrichment efficiency at the tumor site and enables spatiotemporally controlled drug release, further optimizing cancer treatment outcomes. Patent CN116036271A continues the breakthroughs from the previous phase of development, proposing an ultrasound-responsive nanoprodrug system capable of simultaneously releasing nitric oxide gas and camptothecin (Liang et al., 2023). The system covalently links L-arginine and camptothecin via a thioketal bond to form amphiphilic prodrug molecules, which are then combined with a sonosensitizer. Using ultrasonic stimulation, nitric oxide gas and camptothecin can be precisely released at the tumor site, effectively overcoming issues of inaccurate drug ratios and premature leakage encountered in traditional drug delivery methods.

From Patent CN112641725A, CN113694023A, to CN116036271A, the technological path demonstrates the evolution from multiple-trigger responses to dual-drug synergistic therapy. The progressively advancing designs significantly enhance treatment precision, targeting ability, and efficacy, offering more efficient therapeutic strategies for deep-seated and refractory tumors.

#### 3.1.4 Phase IV: the rise of biomimetic and other emerging materials

Regardless of the path, the endpoint of each highlights the innovation and application of biomimetic nanodelivery systems. Biomimetic nanomaterials refer to nanomaterials designed and synthesized by mimicking the structures, functions, or mechanisms found in nature (Gareev et al., 2022; Sarrami-Foroushani et al., 2024). Patent CN114246861A focuses on the preparation method of shear stress-responsive drug-loaded nanoparticles designed for the treatment of atherosclerosis (Li et al., 2022). By synthesizing simvastatin acid (SA) and forming an SA-PEI drug carrier with modified PEI (polyethyleneimine), the system is then electrostatically adsorbed onto the surface of red blood cells (RBCs), resulting in the SA-PEI@RBCs biomimetic drug delivery system. This system takes advantage of the interaction between the negative charge of RBCs and the drug-loaded particles, enabling the specific release of drugs in the high-shear stress environment of AS plaques, thereby enhancing local therapeutic effects and reducing toxicity to healthy tissues. The new physical triggering mechanism not only improves the drug’s biocompatibility and long circulation time but also effectively mitigates the side effects of SA. Patent CN115998711A further advances this technology by proposing a targeted nanodelivery system for reversing tumor drug resistance, which is constructed by physically encapsulating a bionic-type drug delivery carrier with a drug to form a shell-and-core-type nano-complex, and then binding it to the surface of a living cell by electrostatic action (Wang et al., 2023). The carriers used were lipoprotein nanoparticles, which could efficiently encapsulate etoposide platinum and bind to polydimethyldiguanide through electrostatic action to form a self-assembled complex to enhance the drug delivery efficiency. In another approach, patent CN114377139A describes a biomimetic carrier for melanoma chemotherapeutic immunotherapy (Zhang and Li, 2022). This carrier is composed of sulfated skin mucin and thioketone compounds, with the former mimicking extracellular matrix or specific cell characteristics and demonstrating excellent melanoma targeting, while the latter responds to ROS. Furthermore, this carrier integrates immune therapy elements, inducing immunogenic cell death (ICD) of tumor cells through chemotherapy agents such as doxorubicin, thereby activating the body’s immune system and generating a multimodal synergistic anticancer effect. The analysis of these patents reveals the enormous potential of biomimetic nanodelivery systems in responsive design, targeted therapy, and multimodal synergistic treatments. Their innovation and applicability provide broad solutions for precision medicine in the treatment of complex diseases.

Furthermore, the application of MOFs in responsive nanomaterials is also noteworthy. Patent CN116196438A presents a preparation method for an oxidation-responsive nanomedicine formulation (Peng et al., 2023). Using a hydrothermal synthesis approach, ROS-sensitive ligand HOOC-TK-COOH is coordinated with Fe and Cu ions to form double-metal coordination of MOFs nanoparticles (FCT MOFs), within which coked magnesium chlorophyll a (ppa) and *in situ* generated MnO2 (manganese dioxide) are encapsulated. This design enhances the generation of ROS, allowing the nanomedicine to rapidly degrade and release Mn/Fe/Cu ions and ppa, thereby alleviating the tumor hypoxic environment and effectively enhancing the synergistic effects of chemical dynamic therapy and sonodynamic therapy. The innovation of this material not only extends the application of MOFs in responsive nanodrug delivery but also offers a new approach for the precise treatment of complex diseases through multimodal responsive properties.

The analysis of the main and derivative paths clearly reveals the logical development and evolutionary trends of responsive nanomaterial technologies. From early nano delivery systems optimized using traditional methods to the integration of drug delivery and theranostics integration, to the exploration of multiple responses and synergistic treatments, and finally, to the rise of emerging nanodrug delivery systems such as biomimetic systems, the technology continues to advance toward precision, multifunctionality, and intelligence. Throughout this process, the diversification of responsive mechanisms—such as pH, hydrolysis, H₂O₂, and ROS—constantly drives material design to be more efficient and better suited to the complex biological environment. The introduction of MOFs has also showcased their potential in drug delivery, controlled release, and therapeutic synergy. Each stage of technological innovation has expanded and optimized the functionalities of the materials involved. Overall, the evolution of responsive nanomaterial technologies reflects a gradual deepening from basic concepts to multidimensional functional integration, with broad application prospects in cancer treatment, cardiovascular diseases, and theranostics integration. In the future, as biomimetic features and intelligent materials are further combined, and multimodal diagnostic and therapeutic technologies are expanded, responsive nanomaterials are expected to create new possibilities in the field of precision medicine, offering personalized and systematic solutions for the treatment of more complex diseases.

### 3.2 Keyword heatmap analysis

**FIGURE 3:**
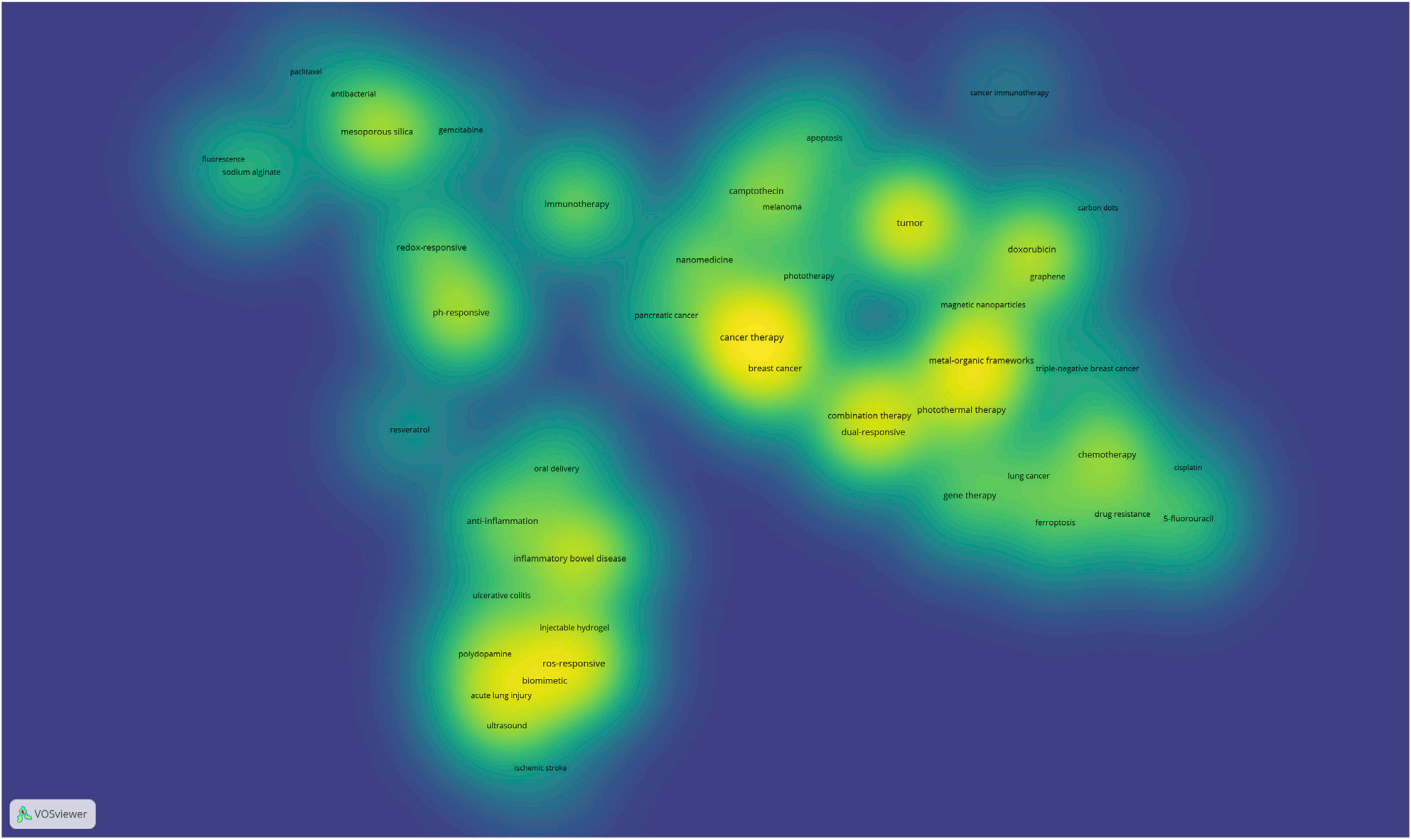
Keyword heatmap of responsive nanomaterials literature.

Through a co-occurrence network analysis of keywords in the literature from 2023 to 2024, the generated heatmap (as shown in Figure 3) not only compensates for the limitations of individual studies but also supplements the key information that was not reflected in the main path analysis due to its inherent lag (Jee et al., 2019). The heatmap reveals additional research directions in the field beyond materials, responsive mechanisms, and preparation methods, such as functional expansion and multimodal applications.

#### 3.2.1 Innovation in emerging materials

Keywords such as “Biomimetic,” “Polydopamine,” and “Injectable Hydrogel” in the heatmap highlight the rapid development trends in biomimetic design and novel materials. This is a point where the scientific literature coincides with and is more nuanced than the technology pathway. For instance, Zhou’s study developed a biomimetic nanodrug delivery system, Rapa@TLNVs, which fuses M2 macrophage membranes with ROS-sensitive thioketal lipids to load rapamycin (Rapa) (Zhou et al., 2024). This system targets atherosclerotic plaques and responsively releases Rapa in a high oxidative stress environment to inhibit inflammation, reduce foam cell formation, and significantly decrease inflammation and lipid deposition in ApoE^−/−^ mice, improving plaque stability. Polydopamine, as a biomimetic coating material, not only enhances the surface stability of nanocarriers but also enables smart drug release through mechanisms such as ROS- and pH-responsive (Liu et al., 2023a; Saraf et al., 2023). Injectable hydrogels, with their three-dimensional network structure resembling the extracellular matrix, demonstrate superior performance in local inflammation and tumor treatment (Jia et al., 2024).

Additionally, novel materials such as mesoporous silica, MOFs, magnetic nanoparticles, and graphene have significantly advanced the functional expansion of responsive nanotechnology. For instance, Wu proposed a multilayer pH-responsive nanodrug delivery system (CDs-CS/HMSNs@DOX), using hollow mesoporous silica nanoparticles as carriers to load doxorubicin (Wu et al., 2024). The system employs non-covalent bonding with chitosan and carboxylated carbon dots to achieve long circulation time, efficient internalization, and precise drug release. In the tumor’s acidic microenvironment, the release of CDs causes a charge reversal, and the expansion of chitosan combined with the exposure of mesopores promotes drug release, while fluorescence recovery enables real-time monitoring. Fang developed a multifunctional targeting nanoplatform (DOX/Au-Apt@ZIF-8) based on the MOF (ZIF-8), combining doxorubicin, the AS1411 aptamer, and gold nanorods to achieve a synergistic anti-tumor effect through chemotherapy, targeting, and photothermal therapy (Fang et al., 2024). ZIF-8 offers excellent pH responsiveness and stability, exhibiting efficient tumor suppression and low toxicity under near-infrared light, providing an innovative solution for breast cancer treatment. Işıklan created a multifunctional composite material (SA-g-PHPM/mGO/EPS) based on graphene and magnetic nanoparticles, which, by combining photothermal and magnetic field responsiveness, significantly enhances the release efficiency of etoposide (Işiklan et al., 2024). These innovative materials not only optimize anti-cancer efficacy but also open new avenues for multi-drug combination therapy and gene delivery.

#### 3.2.2 Intelligent mechanism design and optimization of synergies

The keywords “pH-Responsive” and “ROS-Responsive” in the heatmap highlight the diversification of responsive mechanisms. These mechanisms have long been a research hotspot in the field of responsive nanomaterials. Recent studies have focused on their ability to release anticancer drugs in specific environments to induce other forms of cell death, such as “ferroptosis” (Liu et al., 2023b). Ferroptosis is a form of cell death characterized by iron ion metabolic abnormalities, and it interacts uniquely with the drug resistance of tumor cells (Tian et al., 2024). Tong designed pH-responsive nanocarrier, HDP (hyperbranched polyglycerol), to overcome NRF2-mediated ferroptosis resistance through the co-delivery of sorafenib and siNRF2 (Tong et al., 2024). The HDP-ss nanoplatform synergistically induces ROS generation, iron overload, and GSH depletion, significantly enhancing the ferroptosis effect. In animal experiments, this platform achieved about a 94% tumor suppression rate, providing a new solution to overcome drug resistance in liver cancer therapy.

In addition to single responses, the co-occurrence of keywords like “photothermal therapy,” “combination therapy,” and “dual-responsive” in the heatmap is noteworthy. This pattern aligns with the trends in the technical path, indicating that current research tends to combine various therapeutic approaches. For instance, Pal developed a multifunctional nanoplatform based on copper sulfide (CuS) (Pal et al., 2023). The CuS nanosheets, prepared by precipitating Cu^2^⁺ with H₂S, have near-infrared absorption properties, making them suitable for photothermal therapy. The nanosheet surface is coated with polydopamine and chitosan, targeting breast cancer cells via folic acid and encapsulating drug 5-fluorouracil to achieve a synergistic effect between chemotherapy and photothermal therapy. Additionally, other external physical stimuli, such as “ultrasound,” are widely researched as auxiliary therapeutic technologies in this field. Ultrasound can enhance the accumulation of nanomaterials at target sites and increase drug release rates under external control, improving treatment precision. It has been applied in ultrasound-responsive drug delivery and sonodynamic therapy (Yang et al., 2024; Tang et al., 2024; Chen et al., 2022).

#### 3.2.3 Clinically relevant research

Keywords in the heatmap, such as “Cancer Therapy,” “Immunotherapy,” and “Gene Therapy,” clearly reflect the clinical application trends of responsive nanomaterials in cancer treatment. In particular, studies on refractory cancers such as breast cancer (Ghalehkhondabi et al., 2023; Xu et al., 2023), melanoma (Yan et al., 2024), pancreatic cancer (Rezaei et al., 2023; Tang et al., 2024), and lung cancer (Pei et al., 2024) show that responsive nanomaterials demonstrate significant therapeutic advantages (Xiao et al., 2023; Wang et al., 2024c). For example, the combination of photothermal therapy and chemotherapy significantly improves the precision of drug delivery and therapeutic effects (Sun et al., 2023). When combined with immunotherapy, responsive nanomaterials activate the body’s immune system by inducing ICD, achieving a multimodal synergistic anticancer effect between chemotherapy and immunotherapy (Ashrafizadeh et al., 2023). Additionally, gene therapy, as an emerging cancer treatment approach, uses specific gene fragments or siRNA to inhibit cancer cell growth or enhance the effects of chemotherapy drugs. Responsive nanomaterials provide efficient carriers for gene therapy (Ebrahimi et al., 2023). Nanomaterials not only protect the stability of gene fragments during delivery but also ensure their release in specific tumor environments through responsive mechanisms, thereby improving the precision of treatment (Shamaeizadeh et al., 2024).

The application of responsive nanomaterials in non-oncological diseases has been steadily advancing. For instance, in the treatment of inflammatory diseases such as inflammatory bowel disease (IBD), responsive nanocarriers facilitate the targeted delivery of anti-inflammatory drugs, significantly reducing systemic toxicity while enhancing the therapeutic effect at the local lesions (Long et al., 2024a). For instance, Wang has designed an oral ROS-responsive drug delivery system targeting the inflamed colon in IBD based on the pH and elevated ROS levels at the site of inflammation (Wang et al., 2024a). In the treatment of atherosclerosis, responsive nanomaterials provide a precise and systematic therapeutic approach by alleviating local oxidative stress and inflammation, thus offering a targeted treatment for cardiovascular diseases (Wang et al., 2024b). Furthermore, research into multi-drug resistance has demonstrated significant clinical translation potential. Responsive nanocarriers, using intelligent delivery strategies, not only overcome the barriers posed by drug-resistant cells but also effectively increase the drug concentration at the lesion site (Xiao et al., 2023). These studies offer critical support for disease management and precision medicine.

Compared to the analysis of the technological path, the keyword heatmap provides a more focused view of niche areas, revealing the diversity and innovation in the current research on responsive nanomaterials. Hot topics such as biomimetic design, novel responsive mechanisms, and multimodal synergistic therapy are more precisely represented in the heatmap. At the same time, the heatmap also reflects the broadening clinical applications of responsive nanomaterials. Keywords like “Gene Therapy,” “Immunotherapy,” and “Cancer Therapy” indicate the expanding scope of responsive nanomaterials. Research on the treatment of non-oncological diseases, such as IBD and ischemic stroke, also receives attention, demonstrating its diverse potential in precision medicine. Compared to the main path analysis, the heatmap, by revealing research hotspots in these specialized fields, provides more specific guidance for the future development of responsive nanomaterials in personalized healthcare, complex disease management, and clinical translation.

### 3.3 Progress in clinical trials

In the clinical field, responsive nanomaterials achieve precise drug release and targeted therapy through specific stimulus mechanisms, significantly enhancing therapeutic effects and reducing side effects. These nanomaterials primarily include temperature-responsive, light-responsive, pH-responsive, magnetic field-responsive, and multi-responsive types. For example, PDT, a clinically approved cancer treatment method with over 40 years of history, is applicable to various cancers such as superficial skin lesions, esophageal tumors, and lung tumors. Hundreds of photosensitizers, including porphyrins, dihydroporphyrins, and phthalocyanine derivatives, have been utilized in clinical or preclinical applications (Zeng et al., 2020). The U.S. Food and Drug Administration has approved methyl Aminolevulinate (Picard et al., 2017) and 5-Aminolevulinic Acid (Chi et al., 2020) for the treatment of actinic keratosis, basal cell carcinoma, and squamous cell carcinoma; Radachlorin has been approved by the Ministry of Health of the Russian Federation for the treatment of skin cancer (Shi and Sadler, 2020); and the European Medicines Agency has approved Temoporfin for the palliative treatment of advanced head and neck squamous cell carcinoma (van Doeveren et al., 2018).

Additionally, the pH-responsive nanomaterial Doxil encapsulates doxorubicin within polyethylene glycol-modified liposomes, enabling slow drug release in the acidic tumor microenvironment. Doxil has been approved for the treatment of ovarian cancer, lymphoma, and breast cancer, significantly reducing cardiotoxicity and other systemic side effects (Barenholz, 2012). The magnetic field-responsive nanomaterial Ferumoxytol has been approved by the FDA for the treatment of anemia and shows promising potential in tumor diagnosis and targeted therapy (Long et al., 2024b). Enzyme-responsive nanomaterials, such as silicasome nanoparticles, release drugs in response to tumor-specific enzymes and are currently in the early stages of clinical trials, demonstrating good drug delivery efficiency and safety (Vaghasiya et al., 2021). Multi-responsive nanomaterials combine various response mechanisms; for example, polymeric micelles disassemble to release drugs in response to pH and temperature changes, while lipid nanoparticles facilitate mRNA release in specific intracellular environments for gene therapy or vaccine delivery, accumulating substantial clinical trial data (Pardi et al., 2018). Overall, responsive nanomaterials currently undergoing clinical trials are widely applied in cancer treatment and other disease management through multiple response mechanisms, greatly advancing the development of personalized and precision medicine. With the continuous advancements in nanotechnology and biomedical engineering, it is anticipated that more innovative responsive nanomaterials will enter clinical applications in the future, further optimizing disease treatment regimens and improving patients’ quality of life.

## 4 Limitations and challenges in the field of responsive nanomaterials

Despite the tremendous potential of responsive nanomaterials in the biomedical field, their development and application still face numerous challenges and limitations. Firstly, when designing drug delivery systems, it is imperative to prioritize the biocompatibility, biodegradability, non-toxicity of the nanomaterials, and their ability to be safely cleared from the body (Leite et al., 2015). Individual differences and the heterogeneity and dynamic changes of the tumor microenvironment often significantly affect the responsiveness of the materials and the efficiency of drug release. Concurrently, physiological barriers such as the blood-brain barrier can reduce their precision in delivering drugs to specific tissues or diseases. Taking enzyme-responsive nanomaterials as an example, there are notable physiological and tumor microenvironment differences between animal models and human models in preclinical biosafety evaluations, complicating their clinical translation (Cao et al., 2019). Furthermore, in scenarios involving high doses or long-term use of nanomaterials, there is currently insufficient clinical data to confirm their absolute safety. Additionally, the slow or incomplete metabolic processes of nanomaterials may lead to cumulative damage (Wadhawan et al., 2024). For instance, in phototherapy, the short-term and long-term toxicity risks associated with light-triggered molecular delivery cannot be overlooked. Uncontrolled explosive release of drugs from nanocarriers can often result in acute toxicity issues. Overall, while responsive nanomaterials hold significant promise for advancing personalized and precision medicine, addressing these limitations and challenges is crucial for their successful translation from the laboratory to clinical applications (Fernández and Orozco, 2021).

In addition to the complexity of the nanomaterials themselves, industry regulations and safety considerations also pose critical challenges. The clinical application of responsive nanomaterials must undergo rigorous regulatory review and approval. However, regulatory standards vary across different countries and regions, and existing regulations may not comprehensively address the characteristics of these emerging materials, thereby adding extra difficulties to their commercialization and international promotion. Therefore, future research should focus on selecting nanomaterials that possess excellent properties such as biocompatibility, biodegradability, and non-toxicity for designing drug delivery systems. Furthermore, by conducting more in-depth studies and clinical trials, the translation and application of these materials can be accelerated, thereby truly leveraging the advantages of responsive nanomaterials in precision medicine (Zeng et al., 2022b).

## 5 Conclusion

Responsive nanomaterials, as an intelligent and multifunctional drug delivery and therapeutic platform, have demonstrated significant application potential in the biomedical field. This paper systematically summarizes the technological development trajectory and recent research hotspots in this field through patent and literature data, combined with analysis of main and derivative path and keyword co-occurrence analysis. The study shows that the development of responsive nanomaterials has evolved from single-stimulus responsive delivery systems to theranostics integration and then to multimodal synergistic therapies and biomimetic designs. Early research primarily focused on optimizing the stimulus responsiveness of traditional nanomaterials, such as pH, hydrolysis, and GSH mechanisms, to enhance the precision and targeting of drug delivery. With the introduction of new mechanisms like H₂O₂ and ROS, research gradually shifted toward theranostics integration, as well as multimodal synergistic treatment, driving innovative applications of nanomaterials in disease imaging, targeted therapy, and functional integration. Additionally, biomimetic design, by mimicking natural systems, has improved the biocompatibility and adaptability of materials, expanding their potential applications. The keyword co-occurrence analysis further refines the research hotspots, highlighting key directions such as emerging materials, novel responsive mechanisms, and multimodal synergistic therapy while also demonstrating the broad prospects of responsive nanomaterials in clinical applications.

In the future, the development of responsive nanomaterials should focus on the deep integration of multiple response mechanisms and biomimetic design, aiming to develop nanomaterials with multi-responsiveness. This approach is intended to adapt to complex and variable biological environments, thereby enhancing drug delivery efficiency and reducing side effects. Simultaneously, through in-depth biomimetic design that mimics the structures and functions of natural systems, the biocompatibility and targeting capabilities of nanomaterials can be improved. The combination of such optimization and biomimetic design will lay the foundation for providing more precise and efficient therapeutic solutions.

Furthermore, advanced analytical methods and intelligent technologies play a crucial role in the design and optimization of responsive nanomaterials. Technologies such as big data analysis, machine learning, and artificial intelligence can significantly enhance research and development efficiency and success rates. Through high-throughput screening and predictive modeling, researchers can rapidly identify and optimize materials with specific responsive characteristics. For example, machine learning algorithms can analyze large volumes of experimental data to uncover potential relationships between material structures and their properties, thereby guiding the design and synthesis of new materials. Additionally, constructing comprehensive databases of responsive materials and knowledge graphs can associate and integrate different types of data, promote interdisciplinary collaboration, and discover new research trends and potential synergistic effects. This integration of data resources not only accelerates the material development process but also provides rich references and support for innovative nanomaterial design.

Finally, strengthening the integration of basic research and clinical practice is a key step in promoting the translation of responsive nanomaterials from the laboratory to clinical applications. Establishing standardized production and quality control processes, addressing issues related to large-scale production and regulatory compliance, is fundamental to ensuring the safe and effective application of nanomaterials in actual medical settings. Concurrently, designing personalized responsive nanomaterials by incorporating patients’ genetic, metabolic, and disease characteristics can achieve precision therapy. This approach not only significantly improves therapeutic outcomes but also reduces adverse reactions caused by individual differences, thereby advancing the development of personalized medicine. Moreover, through close collaboration with clinical physicians and medical institutions, nanomaterial technologies can be better applied to actual medical needs, promoting their widespread application in areas such as cancer treatment and chronic disease management. In summary, the future development of responsive nanomaterials requires multifaceted collaborative efforts. By deeply integrating multiple response mechanisms and biomimetic design, applying advanced technologies, integrating data resources, and closely combining basic research with clinical practice, the broad application of responsive nanomaterials in the medical field can be achieved, leading to more precise and efficient therapeutic solutions.

## Abbreviations

ROS, Reactive Oxygen Species; PDT, Photodynamic Therapy; PTT, Photothermal Therapy; MOFs, Metal-Organic Frameworks; HPLC, High-Performance Liquid Chromatography; UPLC, Ultra-Performance Liquid Chromatography; PEG-PLA, Polyethylene Glycol-Polylactic Acid; PEG-PCL, Polyethylene Glycol-Polycaprolactone; EPR, Enhanced Permeability and Retention; ATP, Adenosine Triphosphate; GSH, Glutathione; H2O2, Hydrogen Peroxide; MRI, Magnetic Resonance Imaging; SA, Simvastatin Acid; PEI, Polyethyleneimine; RBCs, Red Blood Cells; PEI, Polyethylenimine.
